# Imeglimin Treatment May Improve Whole-Blood Fluidity and Influence Hemorheology in Patients with Type 2 Diabetes Mellitus: A Post Hoc Analysis of the INFINITY Study

**DOI:** 10.3390/jpm16080405

**Published:** 2026-07-29

**Authors:** Takeshi Osonoi, Shinichiro Shirabe, Miyoko Saito, Mitsuru Hosoya, Norie Watahiki, Nana Shiozawa, Satako Douguchi, Kensuke Ofuchi, Makoto Katoh

**Affiliations:** 1 Naka Kinen Clinic, Naka City 311-0113, Ibaraki, Japan; t-osonoi@kensei-kai.com (T.O.); s-shirabe@kensei-kai.com (S.S.); m-saitou@kensei-kai.com (M.S.); m-hosoya@kensei-kai.com (M.H.); n-watahiki@kensei-kai.com (N.W.); n-shiozawa@kensei-kai.com (N.S.); s-douguchi@kensei-kai.com (S.D.); k-ofuchi@kensei-kai.com (K.O.); 2 Division of Research Promotion, Integrated Research Administration Center, Saitama Medical University, Moroyama-Machi 350-0495, Saitama, Japan

**Keywords:** imeglimin, hemorheology, whole-blood fluidity, erythrocyte deformability, erythrocyte lifespan, type 2 diabetes

## Abstract

**Background**: Patients with type 2 diabetes (T2D) frequently exhibit impaired erythrocyte deformability, which contributes to microvascular dysfunction. We previously reported that imeglimin, a mitochondrial-targeted antidiabetic agent, prolongs erythrocyte lifespan. This study investigated the effects of imeglimin on whole-blood fluidity and its clinical implications in patients with T2D. **Methods**: This post hoc analysis of the INFINITY study included 25 patients with T2D who completed 6 months of imeglimin treatment (2000 mg/day) followed by a 3-month follow-up. Whole-blood fluidity was assessed by measuring whole-blood passage time using a microchannel array flow analyzer (MC-FAN). Hematological parameters, glycemic markers, and vascular indices, including brachial-ankle pulse wave velocity (baPWV) and toe-brachial index (TBI), were also assessed. **Results**: Whole-blood fluidity, assessed by 3-month averages of whole-blood passage time, showed an improvement trend at Months 1–3 (*p* = 0.058) and a significant improvement at Months 4–6 (*p* = 0.016) compared with baseline; this effect was reversed after discontinuation. Erythrocyte lifespan significantly increased by 10–20% during treatment and remained prolonged after discontinuation. Conversely, red blood cell count, hemoglobin, and hematocrit decreased during treatment and returned toward baseline post-discontinuation. At Month 6, baPWV increased, and TBI decreased, both showing reversibility after treatment cessation. **Conclusions**: In this exploratory post hoc analysis, imeglimin treatment was associated with reduced whole-blood passage time measured using the MC-FAN system, suggesting improved whole-blood fluidity in patients with T2D. The clinical and mechanistic significance of this observation requires confirmation in future controlled prospective studies incorporating direct assessments of erythrocyte rheology and microvascular function.

## 1. Introduction

Type 2 diabetes mellitus (T2D) is characterized by various hemorheological abnormalities, including increased blood viscosity, enhanced erythrocyte aggregation, and impaired erythrocyte deformability [1]. These alterations trigger microvascular dysfunction and play a pivotal role in the development and progression of diabetic complications, such as retinopathy, nephropathy, and neuropathy [2,3].

Erythrocyte deformability is a critical determinant of microcirculatory flow. Since capillary diameters are typically smaller than the resting diameter of an erythrocyte, erythrocytes must undergo substantial deformation to traverse the microvasculature [4]. Consequently, reduced erythrocyte deformability leads to increased flow resistance and diminished tissue perfusion [5]. Previous studies have demonstrated that NMNAT patients with T2D exhibit impaired erythrocyte deformability compared with healthy individuals, with proposed mechanisms including hyperglycemia, oxidative stress, and alterations in erythrocyte membrane composition [1,6,7].

Imeglimin is a first-in-class oral antidiabetic agent with a novel mechanism of action that improves mitochondrial function [8,9]. It has been reported to increase the expression of nicotinamide phosphoribosyltransferase (NAMPT) in the salvage pathway within pancreatic β-cells, facilitating the conversion of nicotinamide (NAM) to nicotinamide mononucleotide (NMN). Subsequently, NMN is converted to NAD^+^ by NMN adenylyltransferase (NMNAT), which potentially enhances mitochondrial bioenergetics and insulin secretion [10]. Notably, NMNAT is also expressed in the cytosol of human erythrocytes [11]. In NMNAT-deficient mice, erythrocyte NAD^+^ concentrations are markedly reduced, and the erythrocyte lifespan is significantly shortened—from approximately 60 days in wild-type mice to about 10 days—accompanied by severe morphological abnormalities [12,13]. In a previous analysis of the INFINITY study, we demonstrated that imeglimin prolongs erythrocyte lifespan, which may result in relatively higher HbA1c levels compared to actual glycemic status [14].

Given these biological effects, imeglimin may directly influence erythrocyte function and hemorheology. However, its specific effects on erythrocyte deformability remain to be fully elucidated. Therefore, the present post hoc analysis of the INFINITY Study aimed to evaluate the effects of imeglimin on erythrocyte deformability, a pre-specified secondary endpoint of the original study, together with erythrocyte lifespan, hemorheological parameters, and vascular function.

## 2. Materials and Methods

### 2.1. Study Design

This was a prospective, single-arm, open-label exploratory clinical trial (INFINITY study) conducted at Naka Kinen Clinic, Japan. Participants received imeglimin (TWYMEEG^®^; 1000 mg twice daily) for 6 months. A 2-month pre-observation period was used to establish baseline measurements, and a 3-month post-treatment follow-up period was included to assess potential reversibility of any treatment-related changes. During the observation and follow-up periods, no escalation or initiation of additional antidiabetic agents was allowed, except for continuation of stable doses of metformin or α-glucosidase inhibitors [15]. Erythrocyte deformability was a pre-specified secondary endpoint defined in the original INFINITY Study protocol [15]. Although the primary publication of the INFINITY Study focused on the predefined primary outcomes, the erythrocyte deformability results were not reported in that article. The present manuscript reports the detailed analysis of this pre-specified secondary endpoint for the first time using prospectively collected data from the original study.

### 2.2. Participants

Eligible participants were adult patients with T2D who were either untreated or receiving stable therapy with α-glucosidase inhibitors and/or metformin. The detailed baseline clinical characteristics of this cohort have been reported previously in the primary publication of the INFINITY Study [14]. Briefly, to minimize potential confounding factors affecting hemorheology and vascular indices, the eligibility criteria excluded current smokers, patients with anemia, individuals with an estimated glomerular filtration rate (eGFR) < 45 mL/min/1.73 m^2^, and those receiving antiplatelet or anticoagulant therapy. Female participants were required to be postmenopausal.

**Figure 1:**
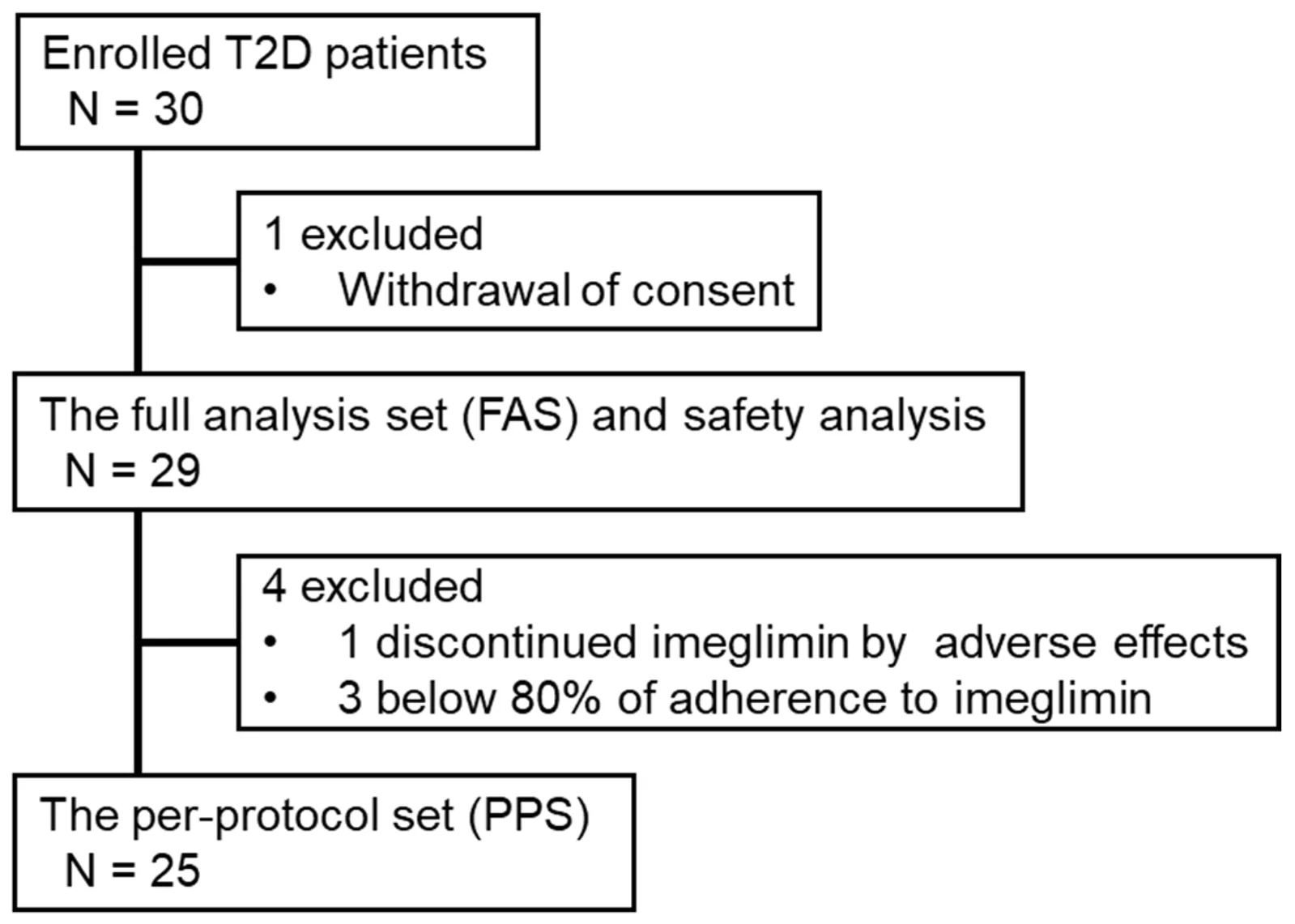
CONSORT-style flow diagram in the INFINITY study adapted from [14].

A total of 30 patients were initially enrolled; however, one patient withdrew consent, resulting in a full analysis set (FAS) of 29 patients. Of these, four patients were excluded due to treatment discontinuation or poor compliance, yielding a per-protocol set (PPS) of 25 patients. The participant flow is shown in Figure 1 (CONSORT-style flow diagram adapted for single-arm trial) [14].

### 2.3. Measurement of Erythrocyte Deformability

Whole-blood fluidity was assessed using a microchannel array flow analyzer (MC-FAN; Optima Inc., Tokyo, Japan), which measures blood passage time through capillary-sized microchannels and provides an integrated assessment of blood rheology, reflecting erythrocyte deformability together with other hemorheological factors. Because erythrocyte deformability is a major determinant of blood passage through microchannels, blood passage time was used as a surrogate index of erythrocyte deformability in the present study.

Heparinized whole-blood samples were introduced into the microchannel array, and the passage of blood cells through the channels was recorded under microscopy. The time required for 100 μL of whole blood to pass through the microchannel array was used as an index of blood fluidity. The blood passage time for each patient was expressed after correction for the passage time of physiological saline. When the passage of 100 μL of whole blood could not be completed because of microthrombus formation or other technical factors, the measurement was regarded as incomplete, and a predefined value of 120 s was assigned in accordance with the MC-FAN measurement procedure [16]. However, the inclusion of such 120 s values across three measurements within a 3-month period may increase data variability. Therefore, in the present post hoc analysis, erythrocyte deformability was evaluated by calculating the mean of the two measurements with the smallest absolute difference within each 3-month interval to reduce the influence of occasional technical artifacts and random short-term fluctuations. For the baseline period, whole-blood passage time was available only at Months −1 and 0 because the measurement was not performed at Month −2 in order to avoid an additional blood draw after informed consent had been obtained. The rate of change from baseline was then determined based on these calculated mean values.

### 2.4. Laboratory Measurements

Fasting blood glucose (FBG), glycated hemoglobin (HbA1c), glycoalbumin (GA), body mass index (BMI), systolic and diastolic blood pressure (SBP and DBP), and pulse rate (PR) were evaluated as absolute values at baseline (0 months), and at 3 and 6 months after initiation of imeglimin treatment, as well as 3 months after treatment discontinuation.

Erythrocyte lifespan was assessed by measuring exhaled carbon monoxide (CO) concentrations using the Carbolizer system (Taiyo Co., Ltd., Osaka, Japan) and was calculated according to the method described by Strocchi et al. [17].

Erythrocyte lifespan (days) = K × hemoglobin (g/mL)/endogenous CO (ppm)

K = 1380 (conversion factor)

Erythrocyte lifespan and other hematological parameters, including red blood cell (RBC) count, and hematocrit, were expressed as percentage changes from baseline based on 3-month interval averages to evaluate temporal trends.

### 2.5. Vascular Measurements

Arteriosclerotic markers were assessed at baseline (0 months), after 6 months of imeglimin treatment, and at 3 months following treatment discontinuation. Arteriosclerosis was evaluated using brachial-ankle pulse wave velocity (baPWV) and toe-brachial index (TBI), measured with a volume-plethysmographic device (BP-203RPEIII; Omron Healthcare Co., Kyoto, Japan). Measurements of baPWV and TBI were performed by trained clinical technicians in a quiet and temperature-controlled clinical measurement room after the patient rested for >5 min in the supine position [18].

### 2.6. Statistical Analysis

The present study was conducted using the PPS, which included patients who completed the study according to the protocol. Data are presented as the mean ± standard deviation (SD) for 25 patients. Statistical significance of changes from baseline was evaluated using the paired *t*-test. Because the present analyses were exploratory post hoc analyses, no adjustment for multiple comparisons was performed. Accordingly, all reported *p*-values were considered nominal. All statistical analyses were conducted using SAS version 9.4 (SAS Institute Inc., Cary, NC, USA). The relationships between changes from baseline to Months 4–6 in erythrocyte deformability or erythrocyte lifespan and other clinical parameters were evaluated using Pearson’s correlation analysis.

## 3. Results

The following analyses were conducted as exploratory post hoc analyses. No adjustment for multiple comparisons was performed; therefore, all reported *p*-values should be interpreted as nominal.

### 3.1. Patient Characteristics and Changes in Laboratory and Clinical Parameters

The analysis included 25 patients (20 males) with T2D who completed 6 months of imeglimin treatment (2000 mg/day) followed by a 3-month post-treatment follow-up period. The baseline demographic and clinical characteristics are summarized in Table 1. The mean age of the participants was 63.5 ± 11.4 years. At baseline (0 months), the clinical characteristics were as follows: BMI, 25.4 ± 3.1 kg/m^2^; HbA1c, 7.5 ± 0.5%; GA, 18.7 ± 2.3%; SBP, 132.1 ± 15.6 mmHg; DBP, 82.9 ± 11.5 mmHg; and PR, 78.1 ± 11.5 beats/min. Regarding vascular indices, the baPWV was 1672.1 ± 412.5 cm/s, and the TBI was 0.835 ± 0.105.

Imeglimin treatment significantly improved glycemic markers in patients with T2D. Compared with baseline (0M), HbA1c decreased 6.9 ± 0.5% at 6 months (*p* < 0.01), and GA decreased 16.5 ± 1.9% (*p* < 0.01). FBG also showed a significant reduction during the 6-month treatment period (*p* < 0.05). After the 3-month discontinuation (9M), all glycemic markers returned to levels similar to baseline. No significant changes were observed in BMI, blood pressure, pulse rate, baPWV or TBI throughout the study.

### 3.2. Effects on MC-FAN-Assessed Whole-Blood Fluidity

**Figure 2:**
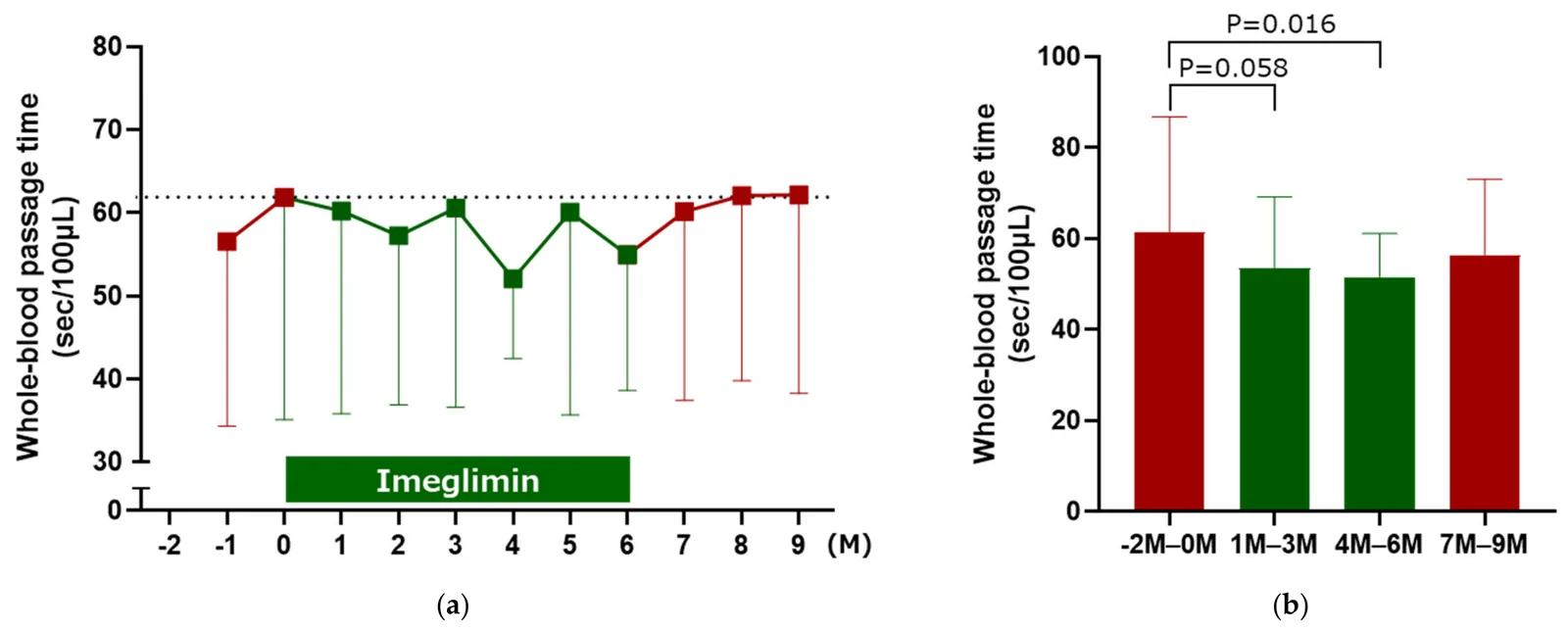
Effects of imeglimin on MC-FAN-assessed whole-blood fluidity. Data are presented as mean ± SD. (**a**) Chronological changes in whole-blood fluidity measured as whole-blood passage time (sec/100 μL). The green bar represents the 6-month imeglimin treatment period. The dashed horizontal line represents the baseline reference (0% change relative to Month 0).; (**b**) Comparison of whole-blood fluidity using 3-month interval averages to mitigate data variability. A significant improvement compared to baseline (−2M to 0M) was observed during the 4–6-month treatment period (*p* = 0.016).

MC-FAN-assessed whole-blood fluidity was evaluated as the time required for 100 μL of heparinized whole blood to pass through the microchannel array. Although initial measurements showed lower values compared to baseline, these differences did not reach statistical significance (Figure 2a). To minimize the impact of random fluctuations and short-term data variability, whole-blood passage time was assessed using means calculated at fixed 3-month intervals. The overall temporal trend observed in the chronological monthly measurements (Figure 2a) was generally associated with that obtained from the 3-month averaged analysis (Figure 2b). Using this approach, whole-blood passage time showed an improvement trend at Months 1–3 of imeglimin treatment (*p* = 0.058) and a significant improvement at Months 4–6 (*p* = 0.016) compared with the pre-treatment period (−2 to 0 months). The mean paired difference in whole-blood passage time between Months 4–6 and the pre-treatment period (Months −2 to 0) was −9.89 s (95% confidence interval, −17.79 to −1.97 s). This effect was reversed after treatment discontinuation, as values returned toward baseline during the 7–9-month follow-up period (Figure 2b).

Sensitivity analyses using (1) the mean of all available measurements within each 3-month interval, (2) the median of the available measurements within each interval, and (3) excluding measurements assigned the predefined value of 120 s all demonstrated the same overall temporal pattern as the primary analysis (Supplementary Table S1). Whole-blood passage time decreased during imeglimin treatment, reached its lowest values during Months 4–6, and returned toward baseline after treatment discontinuation. The average numbers of measurements assigned the predefined value of 120 s per month were 4.0 during the baseline period, 2.7 during Months 1–3, 1.3 during Months 4–6, and 2.7 during Months 7–9.

**Figure 3:**
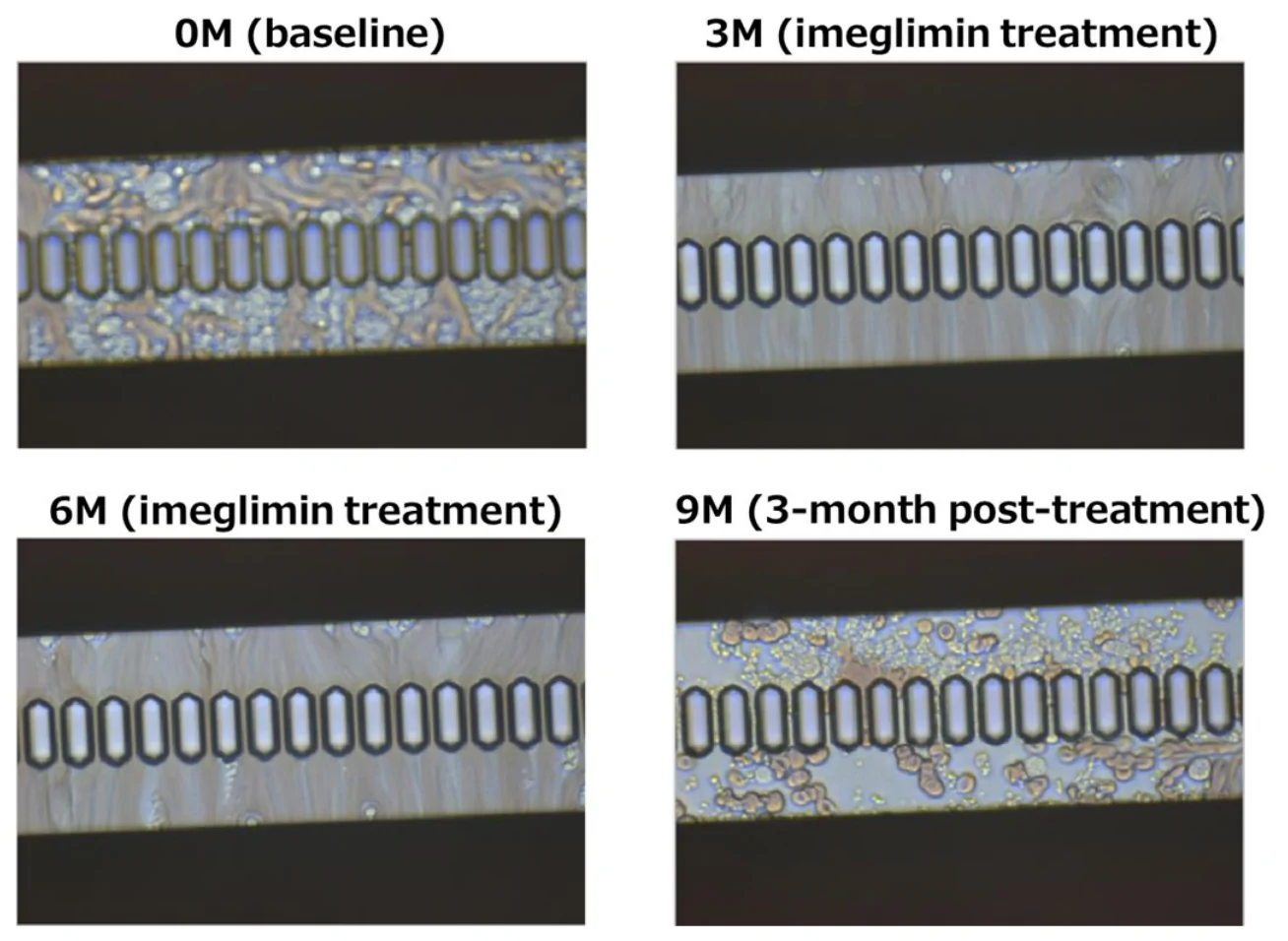
Representative microscopic images of blood flow in the microchannel array. These images capture the passage of blood cells through the microchannel slits (7 µm wide, 30 µm long, and 4.5 µm deep) at baseline (0M), during imeglimin treatment (3M and 6M), and after the follow-up period (9M). Representative images were selected to illustrate typical findings observed at each time point. No quantitative image analysis was performed.

Representative microscopic images obtained using the MC-FAN system are presented in Figure 3 for illustrative purposes to demonstrate the passage of blood cells through the microchannel slits. Representative images are shown at baseline (0M), during imeglimin treatment (3M and 6M), and after the follow-up period (9M). No quantitative image analysis was performed.

### 3.3. Hematological Parameters

**Figure 4:**
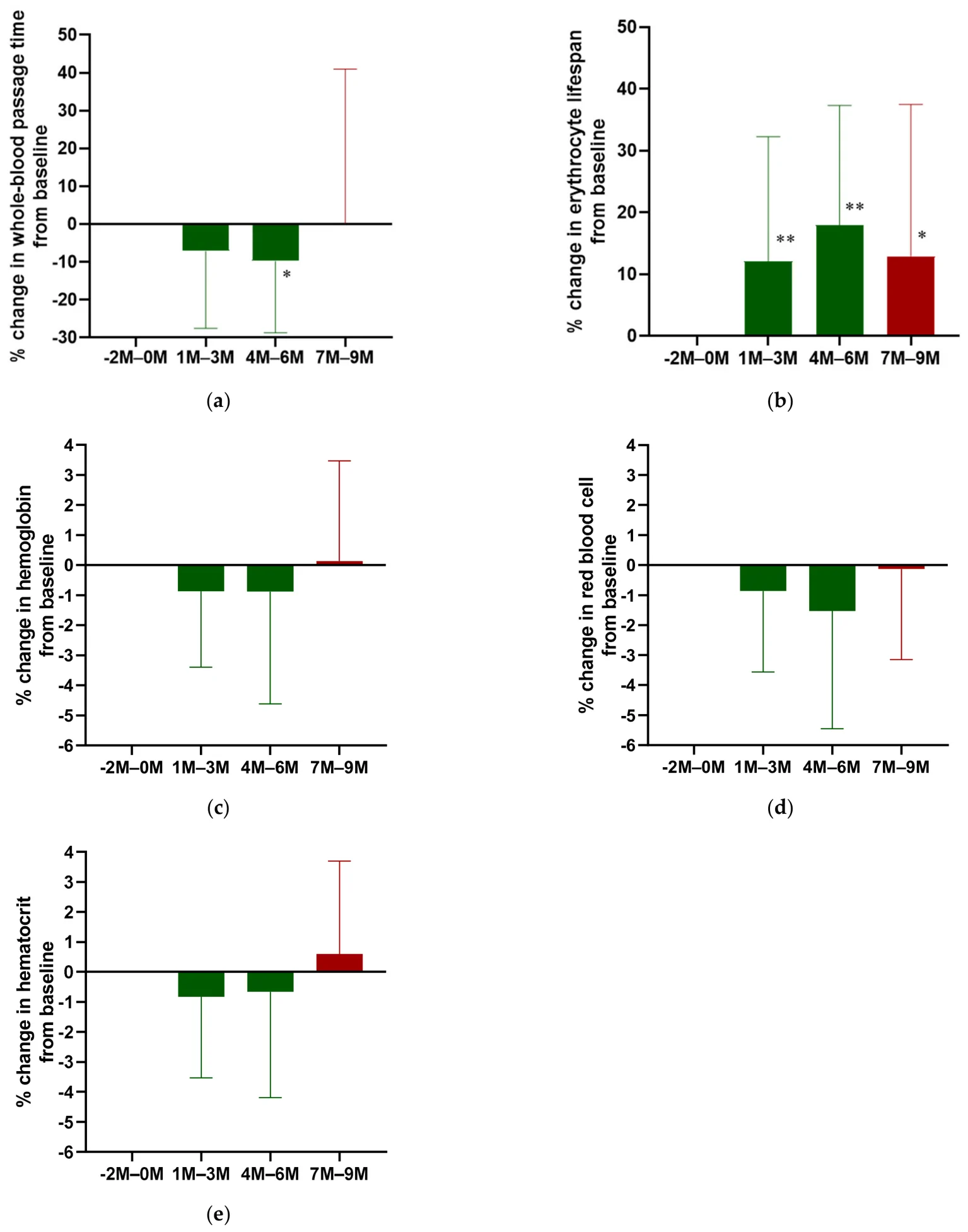
Percentage changes in whole-blood passage time, erythrocyte lifespan, and hematological indices from baseline. Data are presented as mean percentage change ± SD relative to baseline (0M) for 25 patients. The green bar indicates the 6-month imeglimin treatment period. (**a**) Whole-blood passage time: Whole-blood passage time significantly decreased by approximately 10% during the Months 4–6 period, suggesting improved whole-blood fluidity. (**b**) Erythrocyte lifespan: Lifespan significantly increased by 10–20% during treatment and remained elevated during the follow-up period (Months 7–9). (**c**–**e**) Hematological indices: Percentage changes in hemoglobin (**c**), RBC count (**d**), and hematocrit (**e**). These parameters showed a slight, transient decrease during treatment but returned to baseline levels at Month 9. * *p* < 0.05, ** *p* < 0.01 vs. baseline (Months −2 to 0) by paired *t* -test.

Imeglimin treatment exerted distinct effects on erythrocyte dynamics. Evaluation of the percentage change from baseline revealed that blood passage time significantly decreased by approximately 10% at Months 4–6, suggesting improved whole-blood fluidity, before returning to baseline levels after treatment discontinuation (Months 7–9) (Figure 4a). In contrast, the erythrocyte lifespan showed a significant prolongation of 10–20% during the treatment period, and this effect persisted throughout the post-treatment follow-up period (Months 7–9) (Figure 4b). Conversely, the percentage changes in RBC count, hemoglobin concentration, and hematocrit all showed a slight decrease during imeglimin administration, but recovered to baseline levels after discontinuation (Months 7–9) (Figure 4c–e).

### 3.4. Vascular Indices

**Figure 5:**
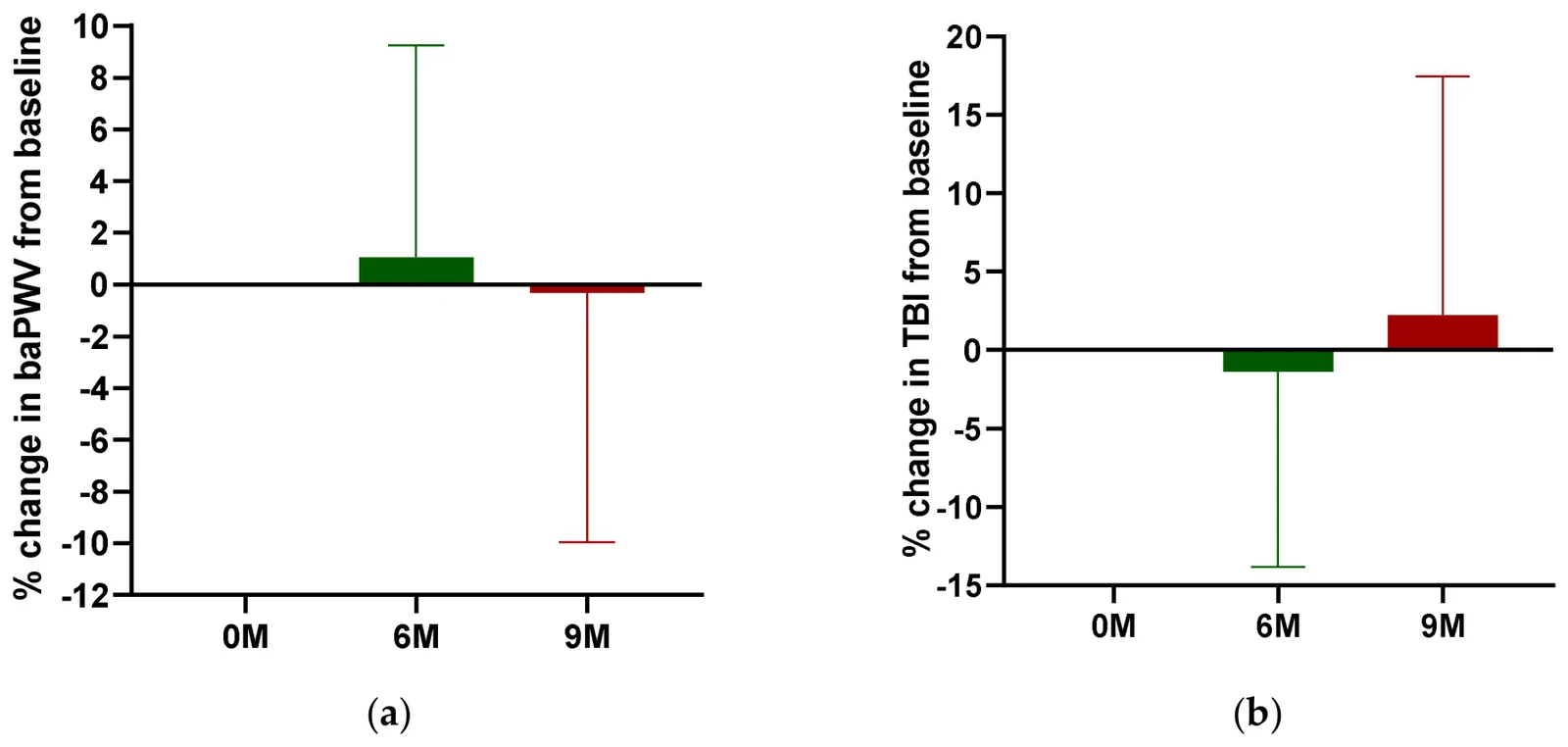
Percentage changes in vascular indices from baseline. Data are presented as mean percentage change ± SD relative to baseline (0M) for 25 patients. The green bar indicates the 6-month imeglimin treatment period. (**a**) Percentage change in baPWV: A slight increase was observed at 6 months, which returned to baseline levels during the follow-up period (9M). (**b**) Percentage change in TBI: A slight decrease was observed at 6 months, followed by recovery toward baseline levels at 9M. No significant differences were observed compared with baseline (0M) at any time point.

To evaluate alterations in vascular function during imeglimin treatment, the percentage changes in baPWV and TBI from baseline were calculated. The baPWV showed a slight increase (approximately 1%) at Month 6 of imeglimin administration, but returned to baseline levels at Month 9, following a 3-month post-treatment period (Figure 5a). Similarly, the TBI showed a marginal decrease (approximately 1.4%) at Month 6 but recovered toward baseline levels by Month 9 (Figure 5b). None of these changes reached statistical significance, and all observations were reversible, returning to baseline values upon treatment cessation.

### 3.5. Correlation Analyses

To evaluate the relationships between changes in whole-blood fluidity or lifespan and other clinical parameters during imeglimin treatment, Pearson’s correlation analyses were performed using the changes from baseline to Months 4–6 (Table 2). No significant correlations were observed between changes in whole-blood fluidity and any of the evaluated parameters, including erythrocyte lifespan, vascular indices (baPWV and TBI), hematological indices, or vital signs. Likewise, changes in erythrocyte lifespan were not significantly correlated with any of the evaluated clinical parameters.

## 4. Discussion

To our knowledge, this is the first study to report a significant association between imeglimin treatment and improved whole-blood fluidity in patients with type 2 diabetes mellitus. In addition, imeglimin treatment was associated with a prolongation of red blood cell lifespan, along with reductions in red blood cell count, hemoglobin levels, and hematocrit, suggesting potential pleiotropic effects on erythrocyte properties and dynamics. In contrast, no statistically significant changes were observed in baPWV or TBI, which are indicators of large-vessel structure and function. Although directional changes were observed during imeglimin treatment and returned toward baseline after treatment discontinuation, these findings should be regarded as exploratory and interpreted with caution.

From a hemorheological perspective, erythrocyte deformability is a major determinant of flow resistance in the microcirculation [19]. Because capillary diameters are smaller than erythrocyte size, a high degree of deformability is required for erythrocytes to efficiently deliver oxygen to tissues [19,20]. In patients with type 2 diabetes mellitus, erythrocyte deformability is known to be impaired due to hyperglycemia, oxidative stress, and alterations in membrane composition, which contribute to the progression of microvascular complications [1,6,7]. In the present study, assessment using a microchannel array flow analyzer (MC-FAN) demonstrated a significant reduction in whole-blood passage time following imeglimin treatment, reflecting improved blood fluidity, with enhanced erythrocyte deformability being one possible contributing factor. Imeglimin has been reported to improve mitochondrial function and increase intracellular NAD^+^ levels in pancreatic β-cells, the liver, and skeletal muscle [9,10]. Notably, human erythrocytes also possess NMNAT, an enzyme involved in NAD^+^ biosynthesis [11,12]. Based on these previous findings, it is conceivable that imeglimin may influence erythrocyte energy metabolism. However, because intracellular NAD^+^, ATP, oxidative stress, and membrane properties were not directly evaluated in the present study, this proposed mechanism remains speculative.

In the present study, baPWV and TBI did not change significantly during imeglimin treatment, and no significant correlations were observed between these vascular indices and whole-blood fluidity. Therefore, no definitive conclusions can be drawn regarding the impact of imeglimin on macro- or microvascular structural function from the present data. The minor, reversible fluctuations observed in these parameters are unlikely to reflect structural alterations in arterial stiffness [21,22]. While it is theoretically possible that alterations in whole-blood fluidity could functionally influence microcirculatory resistance and subsequent wave reflections [23,24], this remains a speculative hypothesis. Given the exploratory nature of this post hoc analysis and the small, uncontrolled study population, these vascular findings should be interpreted with caution.

Furthermore, reductions in erythrocyte count and hemoglobin concentration may contribute to decreased blood viscosity, thereby influencing vascular resistance [23]. In contrast, the increase in mean corpuscular volume (MCV) [14] and the prolongation of erythrocyte lifespan (Figure 4b, [14]) may reflect improvements in erythrocyte membrane properties and metabolic status [25]. The prolongation of erythrocyte lifespan by imeglimin is further supported by experimental evidence demonstrating anti-hemolytic effects mediated by increased NAD^+^ levels in mouse models [12,13]. An extended erythrocyte lifespan may lead to an increased proportion of older erythrocytes, which could contribute to the observed increase in MCV. In the present study, no significant correlation was observed between the prolongation of erythrocyte lifespan and improvements in whole-blood fluidity (Table 2). This finding suggests that erythrocyte lifespan and whole-blood passage time may reflect different physiological aspects of erythrocyte function, although the underlying mechanisms remain unclear.

Taken together, the present findings suggest that imeglimin may improve whole-blood fluidity, thereby contributing to enhanced hemorheology. However, because microcirculatory function was not directly evaluated, its potential effects on the microcirculation remain speculative. Future controlled prospective studies incorporating direct assessments of erythrocyte rheology and microvascular function are warranted to confirm these findings.

This study has several limitations. First, this was a single-arm, open-label exploratory study without a placebo or active comparator group. Therefore, the observed findings should be interpreted as associations observed during imeglimin treatment rather than definitive evidence of causality. In addition, the study was conducted at a single center with a relatively small sample size, which limits the generalizability of the findings. Second, the analyses of the 3-month averages and whole-blood fluidity were exploratory post hoc analyses. No adjustment for multiple comparisons was performed; therefore, all reported *p*-values should be regarded as nominal and interpreted with caution. Accordingly, these findings should be considered hypothesis-generating rather than confirmatory. Third, the assessment of hemorheology was limited to a whole-blood passage time indicator of impaired hemorheology, and more detailed hemorheological parameters, such as erythrocyte aggregation and plasma viscosity, were not evaluated. Accordingly, erythrocyte deformability in the present study should be interpreted as being inferred from whole-blood passage time measured by the MC-FAN system rather than as a direct measurement of erythrocyte membrane deformability. Nevertheless, previous clinical studies have demonstrated significant associations between prolonged whole-blood passage time measured by the MC-FAN system and cardiovascular risk factors or coronary artery disease [26,27,28], supporting the clinical relevance of whole-blood passage time as a surrogate measure of erythrocyte deformability. In addition, the method of averaging the two measurements with the smallest absolute difference within each 3-month interval was introduced in this post hoc analysis to reduce the influence of occasional technical artifacts associated with MC-FAN measurements. Because this approach was not prespecified in the original protocol, it may have introduced analytical bias, and the findings should therefore be interpreted with appropriate caution. However, the consistent trend observed in the unaveraged monthly data partially mitigates this concern. Fourth, the mechanisms underlying the effects of imeglimin on erythrocytes could not be elucidated in this study. Future large-scale and long-term studies integrating these factors are warranted to further clarify the effects of imeglimin on hemorheology and vascular function. Finally, although whole-blood fluidity improved significantly during imeglimin treatment, the clinical significance of this magnitude of change remains uncertain. Because whole-blood passage time is a surrogate marker of hemorheology and microvascular function was not directly evaluated, the present findings should be interpreted as an exploratory association rather than evidence of a direct vascular effect.

## 5. Conclusions

Imeglimin treatment in patients with T2D was associated with reduced whole-blood passage time measured using the MC-FAN system, suggesting improved whole-blood fluidity. The clinical and mechanistic significance of this observation requires confirmation in future controlled prospective studies incorporating direct assessments of erythrocyte rheology and microvascular function.

## Figures and Tables

**Table 1:** Time-course of laboratory and clinical parameters.

Parameter	N	Time (Month) After Imeglimin Administration			
		0	3	6	9
FBG (mg/dL)	25	146.0 ± 23.6	133.6 ± 18.2 *	134.0 ± 24.3 *	154.3 ± 35.2
HbA1c (%)	25	7.5 ± 0.5	7.0 ± 0.5 **	6.9 ± 0.5 **	7.6 ± 0.8
GA (%)	25	18.7 ± 2.3	17.0 ± 2.5 **	16.5 ± 1.9 **	18.8 ± 3.1
BMI (kg/m^2^)	25	25.4 ± 3.1	25.3 ± 3.2	25.3 ± 3.2	25.3 ± 3.0
SBP (mmHg)	25	132.1 ± 15.6	128.9 ± 13.4	132.7 ± 15.1	131.5 ± 12.6
DBP (mmHg)	25	82.9 ± 11.5	83.4 ± 12.1	82.7 ± 11.1	82.8 ± 9.6
PR (beats/min)	25	78.1 ± 11.5	78.0 ± 13.1	79.2 ± 11.4	79.7 ± 13.5
baPWV (cm/s)	25	1672.1 ± 412.5	–	1691.3 ± 446.1	1650.4 ± 368.3
TBI	25	0.835 ± 0.105	–	0.819 ± 0.107	0.847 ± 0.122

Data are presented as mean ± SD. 0M represents baseline; 3M and 6M represent points during imeglimin treatment. Nine months (9M) is a 3-month post-treatment follow-up period. –, not measured. * *p* < 0.05, ** *p* < 0.01 vs. baseline (0M) by paired *t*-test.

**Table 2:** Correlations between changes from baseline to Months 4–6 in whole-blood fluidity or lifespan and other clinical parameters.

Parameter	Whole-Blood Fluidity		Erythrocyte Lifespan	
	r	*p* Value	r	*p* Value
Whole-blood fluidity	–	–	0.219	0.292
Erythrocyte lifespan	0.219	0.292	–	–
TBI	0.278	0.179	0.075	0.723
baPWV	0.077	0.714	0.260	0.209
RBC count	0.351	0.085	0.272	0.189
Hemoglobin	0.276	0.182	0.246	0.235
Hematocrit	0.366	0.072	0.279	0.177
SBP	0.071	0.735	0.046	0.829
DBP	0.302	0.142	0.352	0.084
PR	0.242	0.244	0.127	0.544

Data are presented as Pearson’s correlation coefficients (r) and corresponding *p* values. Correlation analyses were performed using changes from baseline to Months 4–6 for each participant (*n* = 25).

## Data Availability

The data supporting the findings of this study are available from the corresponding author upon reasonable request. Due to ethical restrictions and participant confidentiality, access to the data may be limited and will be provided in accordance with institutional guidelines and applicable data-sharing policies.

## Supplementary Materials

The following supporting information can be downloaded at: https://www.mdpi.com/article/10.3390/jpm16080405/s1. Table S1: Sensitivity analyses of MC-FAN-assessed whole-blood passage time using alternative summary methods and exclusion of incomplete measurements.

## Abbreviations

The following abbreviations are used in this manuscript:

baPWV	Brachial-ankle pulse wave velocity
BMI	Body mass index
CO	Carbon monoxide
DBP	Diastolic blood pressure
FAS	Full analysis set
FBG	Fasting blood glucose
GA	Glycoalbumin
HbA1c	Hemoglobin A1c
MCV	Mean corpuscular volume
NAM	Nicotinamide
NAMPT	Nicotinamide phosphoribosyltransferase
NMN	Nicotinamide mononucleotide
NMNAT	Nicotinamide mononucleotide adenylyltransferase
PPS	Per-protocol set
PR	Pulse rate
RBC	Red blood cell
SBP	Systolic blood pressure
SD	Standard deviation
T2D	Type 2 diabetes
TBI	Toe-brachial index
