# Supplementary material for: Imeglimin Treatment May Improve Whole-Blood Fluidity and Influence Hemorheology in Patients with Type 2 Diabetes Mellitus: A Post Hoc Analysis of the INFINITY Study
Source: J Pers Med. 2026 Jul 29;16(8):405. doi: 10.3390/jpm16080405 (PMC13514736; doi:10.3390/jpm16080405)
Supplement: Supplementary file 1 [file jpm-16-00405-s001.zip › jpm-4316519-supplementary.pdf]

Journal of
Personalized Medicine

Supplementary Table S1. Sensitivity analyses of MC-FAN-assessed whole-blood passage time using alternative summary methods and exclusion of incomplete measurements.

Analysis		Baseline	1–3M	4–6M	7–9M
**Primary analysis** (mean of the two measurements with the smallest absolute difference)	Mean	61.5	53.6	51.6	56.4
	SD	25.2	15.6	9.5	16.7
	p	–	0.058	0.016	0.338
**Sensitivity analysis 1** (mean of all available measurements within each 3-month interval)	Mean	61.5	59.3	55.7	61.3
	SD	25.2	17.3	12.2	18.5
	p	–	0.636	0.185	0.962
**Sensitivity analysis 2** (median of the available measurements within each interval)	Mean	61.5	54.6	52.3	57.9
	SD	25.2	15.4	9.6	16.8
	p	–	0.094	0.020	0.482
**Sensitivity analysis 3** (excluding measurements assigned the predefined value of 120 seconds)	Mean	56.5	55.0	54.4	56.9
	SD	15.2	9.4	9.8	11.0
	p	–	0.529	0.432	0.794

Whole-blood passage time is expressed in seconds. Values are presented as mean ± SD unless otherwise indicated. *p* values were calculated using paired *t*-tests versus the baseline period.
